# Supplementary material for: Eliminating the missing cone challenge through innovative approaches
Source: J Struct Biol X. 2024 May 31;9:100102. doi: 10.1016/j.yjsbx.2024.100102 (PMC11220036; doi:10.1016/j.yjsbx.2024.100102)
Supplement: Supplementary Data 1 [file mmc1.docx]

**Eliminating the Missing Cone Challenge through Innovative Approaches**

Cody Gillman^1,2^, Guanhong Bu^1^, Emma Danelius^1,3^, Johan Hattne^1,3^, Brent Nannenga^4,5^, Tamir Gonen^1,2,3,6, *^

1 Department of Biological Chemistry, University of California, Los Angeles, CA, USA.

2 Molecular Biology Institute, University of California, Los Angeles, Los Angeles, CA 90095, USA.

3 Howard Hughes Medical Institute, University of California, Los Angeles CA, USA.

4 Chemical Engineering, School for Engineering of Matter, Transport and Energy, Arizona State University, Tempe, AZ, USA.

5 Center for Applied Structural Discovery, Biodesign Institute, Arizona State University, Tempe, AZ, USA.

6 Department of Physiology, University of California, Los Angeles, CA, USA.

* To whom correspondence should be sent T.G. tgonen@g.ucla.edu


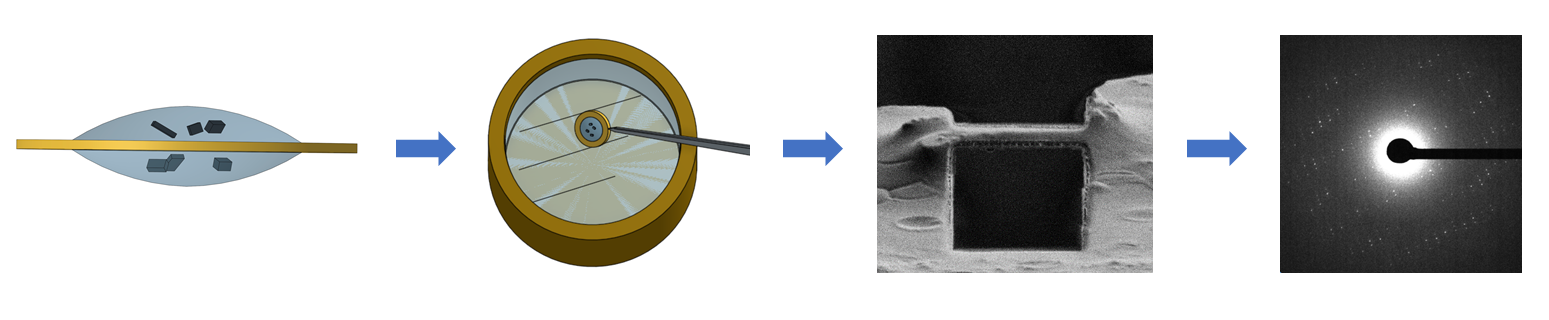


**Supplemental Figure 1:** **An illustration of support free grids with suspended crystals and the sample preparation method for MicroED.** From left to right – suspended crystals on a support free grid viewed from the side; freezing of said grid using liquid nitrogen or ethane for cryoEM; FIB milling to generate thin lamellae for MIcroED; an example of a raw diffraction pattern obtained from the MicroED data set.


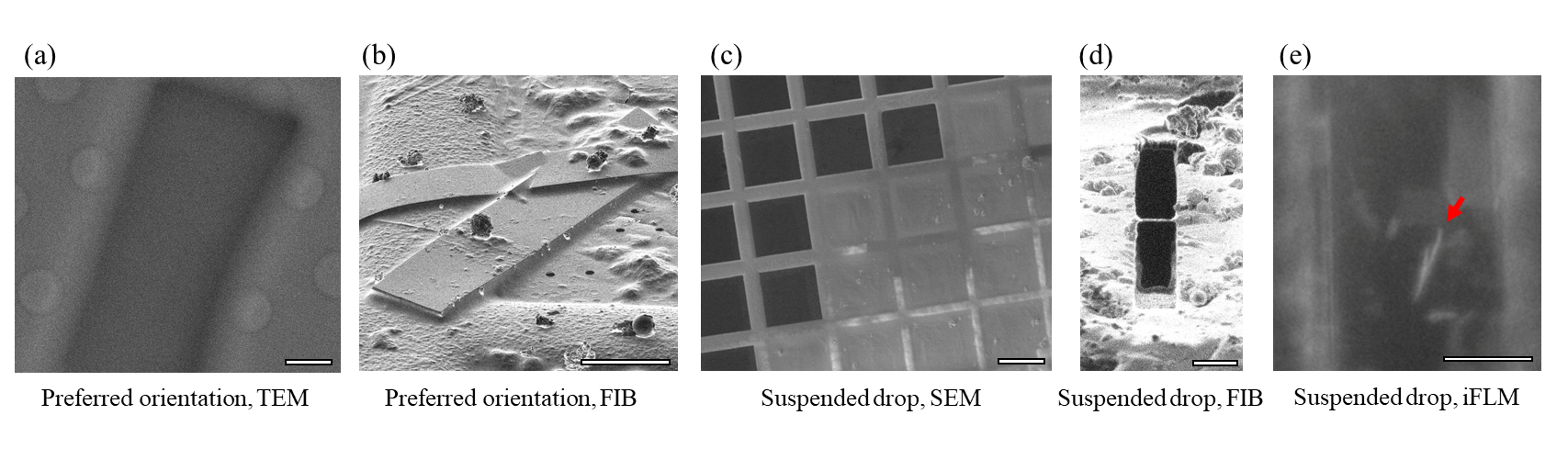


**Supplemental Figure 2:** **Example of the flat crystals preferentially oriented on carbon support grids and the milling support free grids with suspended crystals** (a) TEM image and (b) FIB image of plate crystals showing preferred orientation on the support film of TEM grid. (c) SEM image of suspended drop on the support-free TEM grid in the cryo-FIB/SEM. (d) FIB image of lamella after milling a randomly oriented crystal in the suspended drop. (e) Identification of crystals in suspended drop from the fluorescence light microscope integrated (iFLM) in the cryo-FIB/SEM, other orientations of plate crystals shown in the red arrow. Scale bars represent 2 µm in (a); 10 µm in (b), (d), (e); and 200 µm in (c).
